# Southern Surgical and Gynæcological Society of Baltimore

**Published:** 1892-01

**Authors:** William S. Gardner

**Affiliations:** Secretary; 712 N. Howard St.


### GYNECOLOGICAL AND OBSTETRICAL SOCIETY OF BALTIMORE. NOVEMBER MEETING.

---

The president, Dr. William E. Moseley, in the chair.

Dr. John Morris gave an address entitled "A Parting Word Upon Obstetrics."

"I began the practice of obstetrics forty-six years ago, and for the first four years kept a record of my cases. The first year I attended thirty-five cases. I was associated with Dr. Hintze, who at that time had a very extensive general practice, and who was very often called to assist midwives in their troublesome cases. I kept a careful record of my first two hundred cases, but after that I abandoned the record, a fact which I have since very much regretted.

"My first case was a very unfortunate one. I attended the patient in my student days. This woman was in the country, and was in labor three days. At the end of that time I sent for Dr. Hintze, who delivered her with the crochet. On account of the long impaction of the head, the whole of the anterior wall of the vagina sloughed away. The woman is still living, but so much tissue was destroyed that it was quite impossible to close up the opening, and all these years the urine has been passing from her as rapidly as secreted.

"My second case was a black woman who had a prolonged labor. I had never seen the forceps used, but tried to put them on and failed. After a while the child was born without any artificial assistance.

"One of my great difficulties in my first cases was to find the cervix. I had never had any practical instruction in obstetrics, and did not know that in the first stage before much dilatation that the os is usually found far back against the sacrum. Among other things that I think I have learned, is how to shorten labor. One of the best means of accomplishing this is by external pressure. I learned that from my master, Dr. Hintze. Another was to pass the cervix around the occiput; and I found that these two shortened labor very considerably. I think I acquired the art of preserving the perineum. I believe in keeping the head under control and not allowing it to be delivered too rapidly. In Ireland I learned how to preserve the perineum when using forceps. The secret is simply to change the axis of traction as the head comes to the perineum first upwards, perpendicular to the bed, and then carrying the handles far over on to the abdomen of the mother.

"I have found that midwifery is underrated in the profession; but I am convinced that in no branch is there greater opportunity to display skill and judgment. This branch is esteemed much more highly now than formerly.

"Formerly in conditions of rigid cervix it was the practice to bleed. I have done it many times, but it would not be tolerated now.

"I am convinced that hot water injections will assist in relaxation.

"I have no faith in belladonna.

"I have been fortunate in not seeing any cases of hemorrhage. I believe external pressure used during labor will prevent post partum hemorrhage. For the first ten years I used ergot in nearly every case during the second stage; but have not used it now for fifteen years. In cases of delayed labor I now prefer the forceps to ergot.

"The crochet has gone out of use, but formerly it was used frequently. Often the woman was injured and not infrequently the doctor's fingers suffered. Dr. Hintze had a glove to protect his fingers.

"We had at that time no chloroform, and often in transverse positions the woman would die undelivered because it was not possible to turn and deliver. I have not habitually used anæsthetics except in forceps cases. I have thought that they prolonged the labor but I always use chloroform when any force is to be resorted to.

"I have never used the binder because I could never see the philosophy of it. It will not stay in position and it is absurd to think it controls hemorrhage. The only good that I could ever see that it accomplished was to please the woman.

WHEN TO USE FORCEPS.

"Always use forceps when labor is delayed in the second stage. The old forceps were a much weaker instrument than the ones constructed on the Tarnier principle. I think the Tarnier forceps the greatest advance in obstretrics in my time.

"In placenta previa and in abortion we formerly used a tampon made of a handkerchief, rags, cotton or anything that could be had. These tampons were dirty and dangerous. Later I have used only the colpruryuulur. It assists to dilate the os as well as being the most efficient tampon. It is clean and harmless.

"Opium is the best thing to relieve pain in labor. It does not arrest labor. When the os is dilated it increases the contractions.

Dr. F. E. Chatard exhibited at the society the obstetrical instruments used by his grand-father 1810—1840, and also those used by his father 1835—1875. He stated that he had used external pressure with apparent good effect.

Dr. Wilmer Brinton stated that external pressure was used by primitive people. He thought that in rigid os he had gotten good results from the administration of chloral in fifteen grain doses every fifteen minutes until three doses were given, as recommended by Playfair. But the number of cases in which he had given chloral was small.

Dr. G. Lane Taneyhill had used chloral per anum with great satisfaction in three cases. In less than an hour the os had been considerably dilated, and delivery was effected in each case in three hours, other remedies having failed. He had learned this treatment from our learned fellow-member, Dr. Williams—he uses 30 grs. chloral in milk.

Dr. P. C. Williams thought it was very important to consider agents to relax the parts. Chloral in 40 to 60 grain doses per anum had given good results, but sometimes it, as well as chloroform, fails to completely relax the cervix.

In his earlier experience he had encountered many cases of post partum hemorrhage, but since he had made use of a practice that is condemned by most obstetricians, that of giving ergot before chloroform, he had not had a single case of hemorrhage. He had seen no harm result from the practice but thought he had in this way shortend the labor. The objection to morphine to relieve pain is that it nauseates badly afterwards. Chloral must be pushed to get good effects. The objection to it is that sometimes it leaves the patient more or less delirious, and may seriously depress the heart if given too frequently.

Dr. William S. Gardner had used chloral in fifteen grain doses repeated every fifteen minutes in a series of cases, and found that while the patients had very little relief from pain, that a large percentage of them would be made sick at the stomach, and the discomfort caused by the disagreeable taste of the drug and by the vomiting following its use, more than counterbalanced the little good it did, and its use in this way was abandoned.

He gives it frequently for the relief of false labor pains: A dose of 30 grs. will almost invariably relieve the pains and put the woman to sleep.

Dr. Wm. P. Chunn had used chloral a number of times but could get no positive evidence of its value, but it does not seem to obtund the pain. If opium will do this it might be advisable to use it.

Dr. L. E. Neale was surprised that a discussion as to the value of chloral should be brought up. He thought that the time for discussion of that subject had passed. Whether it would act more efficiently by the rectum or by the stomach he did not know, but he thought 60 grains too large a dose and would be afraid to use that much as an ordinary dose by the mouth. The remarks were entirely too general to admit of special discussion.

WILLIAM S GARDNER, Secretary.

712 N. Howard St.

